# Use of Belatacept as Alternative Immunosuppression in Three Renal Transplant Patients with *De Novo* Drug-Induced Thrombotic Microangiopathy

**DOI:** 10.1155/2013/260254

**Published:** 2013-10-01

**Authors:** Federico Cicora, Marta Paz, Fernando Mos, Javier Roberti

**Affiliations:** ^1^Renal Transplantation, Hospital Alemán, Pueyrredón 1640, C1118AAT Buenos Aires, Argentina; ^2^Foundation for Research and Assistance in Renal Disease (FINAER), Calle 503 No. 1947, CP B1897FYU, Gonnet, Buenos Aires, Argentina

## Abstract

Thrombotic microangiopathy (TMA), a severe complication of renal transplantation, is a pathological process involving microvascular occlusion, thrombocytopenia, and microangiopathic hemolytic anemia. It generally appears within the first weeks after transplantation, when immunosuppressive drugs are used at high doses. *De novo* TMA may also be drug-induced when calcineurin inhibitors or proliferation signal inhibitors are used. We report three cases of *de novo* drug-induced TMA in renal transplant patients who were managed by replacing calcineurin inhibitors or proliferation signal inhibitors with belatacept, a primary maintenance immunosuppressive drug, which blocks the CD28 costimulation pathway, preventing the activation of T lymphocytes. To identify the cause of TMA, we ruled out HUS, hepatitis C serology, HIV serology, parvovirus B19, cytomegalovirus, anti-HLA antibodies, and prolonged activated partial thromboplastin time. We suspect that the TMA was caused by the calcineurin inhibitors or proliferation signal inhibitors. Belatacept treatment was initiated at a dose of 10 mg/kg on days 1, 5, 14, 28, 60, and 90; maintenance treatment was 5 mg/kg once a month for 1 year. Belatacept, in combination with other agents, prevented graft rejection in three patients.

## 1. Introduction

Thrombotic microangiopathy (TMA), a severe complication of renal transplantation, is a pathological process that involves microvascular occlusion, thrombocytopenia, and microangiopathic hemolytic anemia. [1–3]. When renal lesions are more common, the clinical entity is defined as hemolytic uremic syndrome (HUS), and when brain lesions prevail, it is termed thrombotic thrombocytopenic purpura [2]. Posttransplant TMA can occur *de novo *or may be recurrent if the patient's end-stage renal disease involved HUS [2]. The incidence of *de novo* TMA in renal transplantation is reportedly 0.8% to 3.3% [2, 4]. It generally appears within the first weeks after transplantation, when immunosuppressive drugs are used at high doses [2]. Although the exact pathogenesis of TMA is not fully understood, it has been found that *de novo* TMA may be drug-induced when calcineurin inhibitors (CNIs) or proliferation signal inhibitors (PSIs) are used [3–6]. Other risk factors include ischemia-reperfusion injury, viral infections, and antibody-mediated rejection [4].

If TMA is not treated, it can lead to graft loss or renal cortical necrosis [4]. Typical strategies for treatment of *de novo* TMA include reduction or withdrawal of CNI, switching from CNIs to PSIs, such as sirolimus, reducing the CNI, and then restoring it after clinical recovery [2, 6]. Other suggested therapies include plasmapheresis and the use of intravenous immunoglobulin (IVIg) in combination with steroids, rituximab, or eculizumab [3, 7, 8]. Choosing the right immunosuppressive therapy strategy represents a challenge because both CNIs and PSIs have been associated with TMA, but good results have also been reported with use of these agents [2, 9, 10]. To our knowledge, the use of belatacept has been reported only once previously [10]. Belatacept is an immunosuppressive drug that blocks the CD28 costimulation pathway, inhibiting T-lymphocyte activation [11, 12]. Here, we report three renal transplant patients with *de novo* drug-induced TMA who were managed with belatacept as an alternative immunosuppressive agent.

## 2. Case Reports

### 2.1. Patient 1

**Figure 1:**
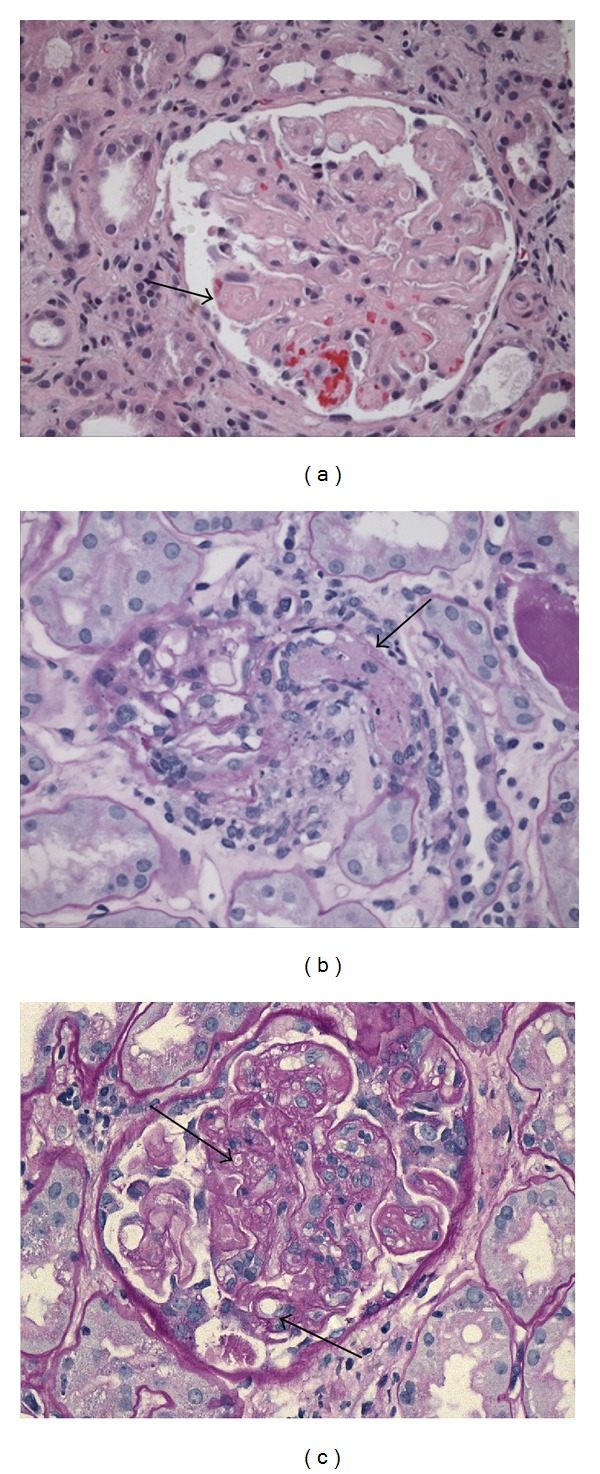
Light micrographs showing TMA. (a) Patient 1: H&E 20x: glomerulus with consolidated appearance caused by swelling of endothelial cells (endotheliosis). (b) Patient 2: PAS, 20x: glomerulus with an arteriole occluded by a thrombus. (c) Patient 3: PAS, 40x: mesangiolysis and double contours.

A 33-year-old male received a living-relative renal transplant; his mother was the donor. When the patient was 8 months old, he had suffered from typical HUS. Induction therapy consisted of basiliximab on day 0, and because the graft showed delayed function, antithymocyte globulin at 1.25 mg/kg daily was administered for 6 days. Maintenance therapy consisted of tacrolimus, MPA, and prednisone; ganciclovir was used for CMV prophylaxis. On postoperative day (POD) 150, to prevent toxicity related to CNI, tacrolimus was discontinued and replaced with everolimus at 1.50 mg daily with a goal trough of 3–8 ng/mL, and MPA was administered at 1440 mg daily. On POD 240, his creatinine level was 154.70 *μ*mol/L (1.75 mg/dL). On POD 330, the patient became intolerant of MPA and developed diarrhea; the drug was withdrawn, and prolonged-release tacrolimus at 7 mg was introduced. On POD 740, the patient was admitted with deteriorating renal function and creatinine of 291.72 *μ*mol/L (3.3 mg/dL). A biopsy confirmed mesangiolysis and interstitial fibrosis and tubular atrophy (IFTA) Grade I (Figure 1(a)). C4d staining was negative, no glomerulitis or capillaritis was present, and detection of donor-specific antibodies (DSA) by Luminex was negative. At the time of TMA diagnosis, laboratory tests showed the following values: creatinine 247.52 *μ*mol/L, hemoglobin 141 g/L, platelet count 145000/mm^3^, tacrolimus trough level 10.4 ng/mL, everolimus 7.6 ng/mL, total bilirubin 25.65 *μ*mol/L, and unconjugated bilirubin 7.86 *μ*mol/L, and no schistocyte was detected. Recurrence of HUS and other possible causes were ruled out. Tacrolimus and everolimus were discontinued, and belatacept was introduced, beginning at 10 mg/kg on days 1, 5, 14, 28, 60, and 90; maintenance treatment was 5 mg/kg once a month for 1 year. Other immunosuppressive drugs included prednisone at 4 mg daily and MPA at 1440 mg daily. On POD 800, 60 days after the TMA diagnosis, his creatinine was 194.48 *μ*mol/L (2.2 mg/dL), and a repeat biopsy showed no TMA.

### 2.2. Patient 2

A 55-year-old female, with unknown primary renal disease, who had been on hemodialysis for 5 years, underwent a transplant with her sister as the donor. After induction therapy with basiliximab at 20 mg on days 0 and 4, she showed good diuresis and a decrease in her urea and creatinine levels. Immunosuppression was achieved with corticosteroids, tacrolimus at 0.15 mg/kg to achieve a trough level of 6–10 ng/dL, and MPA at 1440 mg/day; prophylaxis against infection consisted of ganciclovir and trimethoprim/sulfamethoxazole. At the time of discharge on POD 5, her creatinine was 137.02 *μ*mol/L (1.55 mg/dL). On POD 15, the patient's creatinine was 300.56 *μ*mol/L (3.4 mg/dL). A biopsy confirmed Banff Ia cellular rejection; treatment with three pulses of methylprednisolone resulted in a decreased creatinine level. Between POD 15 and 30, the patient's course was complicated by deep vein thrombosis, a hematoma on the abdominal wall, and a urinary fistula. On POD 20, she was admitted with pain in the area of the graft, an increased creatinine level of 548.08 *μ*mol/L (6.2 mg/dL), and Banff IIa cellular rejection. Antithymocyte globulin was administered at 1.25 mg/kg daily for 10 days. On POD 25, her creatinine increased again, prompting us to perform another biopsy that confirmed acute TMA in the initial phase, mild ATN, and IFTA Grade I (Figure 1(b)). C4d staining was negative, there was no sign of glomerulitis or capillaritis, and detection of anti-HLA antibodies by Luminex was negative. At the time of TMA diagnosis, test values were as follows: creatinine 548 *μ*mol/L (6.19 mg/dL), platelet count 55000/mm^3^, hemoglobin 91 g/L, tacrolimus trough level 15.7 ng/mL, lactate dehydrogenase (LDH) 167 IU/L, and total bilirubin 5.98 *μ*mol/L, and no schistocytes were found. Plasma exchange sessions and administration of IVIg at 100 mg/kg after plasmapheresis were performed. Other possible causes were ruled out. Belatacept was started at 10 mg/kg on days 1, 5, 14, 28, 60, and 90; maintenance treatment was 5 mg/kg once a month for 1 year. Additionally, the patient continued to receive MPA and prednisone. On POD 90, a biopsy showed no sign of rejection or TMA, her creatinine level was 90.17 *μ*mol/L (1.02 mg/dL), and she did not develop any serious adverse event.

### 2.3. Patient 3

A 44-year-old male who had been on hemodialysis for 2 years underwent a living-relative transplant with his brother as the donor. His baseline renal disease was unknown. Induction therapy consisted of thymoglobulin and maintenance immunosuppression corticosteroids, tacrolimus at 0.15 mg/kg to achieve a trough level of 6–10 ng/dL, and MPA at 1440 mg/day; prophylaxis against infection consisted of ganciclovir and trimethoprim/sulfamethoxazole. At the time of discharge on POD 4, his creatinine was 125.53 *μ*mol/L (1.42 mg/dL). On POD 8, a biopsy performed because of a creatinine increase showed Banff Ia cellular rejection, which was treated successfully with three pulses of methylprednisolone. On POD 13, a control biopsy showed mesangiolysis and double contours (Figure 1(c)). C4d staining was negative, there was no sign of glomerulitis or capillaritis, and detection of anti-HLA antibodies by Luminex was negative. Associated causes were ruled out, and it was suspected that the TMA was drug-related. At the time of the TMA diagnosis, no DSA was detected, and laboratory test values were as follows: creatinine 178.57 *μ*mol/L (2.02 mg/dL), platelet count 218000/mm^3^, tacrolimus trough level 6.9 ng/dL hemoglobin 88 g/L, LDH 230 IU/L, total bilirubin 8.20 *μ*mol/L, and haptoglobin 1.06 g/L, and no schistocytes were found. Tacrolimus treatment was suspended, and belatacept was initiated at 10 mg/kg on days 1, 5, 14, 28, 60, and 90; maintenance treatment was 5 mg/kg once a month for 1 year. The creatinine level decreased. On POD 31, a control biopsy showed Banff IIa cellular rejection that was treated with ATG. Administration of belatacept continued as planned. On POD 40, a biopsy showed no sign of rejection or TMA; his creatinine level was 122.88 *μ*mol/L (1.39 mg/dL).

## 3. Discussion

Although mortality associated with TMA has decreased since the introduction of plasma exchange therapy, it can still be a life-threatening condition [13]. In patients who have undergone renal transplantation, the incidence of TMA is higher than in the general population and can lead to graft loss [14], reaching as high as 50% [6]. The most common factors for developing posttransplant *de novo* TMA are associated with deceased-donor transplantation, but TMA also occurs in living-donor transplantation as a result of CMV, HIV, and therapy with specific drugs, among other factors [6]. At our center, between 2009 and 2012, 118 renal transplants were performed at our center, and the incidence of TMA in renal transplant patients was 3.4%.

Drug-induced TMA and AMR as a predisposing factor for TMA should be worked up as differential diagnoses because the two entities, which are difficult to distinguish, require different therapeutic strategies. C4d staining of peritubular capillaries is typical in AMR [2] and can be used as a diagnostic criterion. Additionally, the detection of donor-specific anti-HLA antibodies and the presence of glomerulitis and capillaritis in the biopsy are diagnostic markers of AMR. In our cases, to be able to conclude that TMA was drug-induced, we ruled out possible associations with HIV, hepatitis C, CMV, parvovirus B19, anti-HLA antibodies, and prolonged activated partial thromboplastin time. However, it is important to note that recurrent HUS is difficult or even impossible to rule out. Two of the patients did not show signs of hemolytic anemia, only creatinine level increases, which are common in posttransplant TMA, when diagnosis can be confirmed by biopsy only.

The effects of immunosuppression on drug-induced TMA remain to be determined, and guidelines have not yet been established [14]. Reported options to treat drug-induced TMA include withdrawal of the offending drug and replacement with another, such as cyclosporine, sirolimus, or everolimus [3]; usually, this is accompanied by plasma exchange or infusion [2, 3, 5, 6, 8, 10]. More recently, eculizumab in TMA associated with AMR has been suggested for prophylaxis or as an alternative treatment [2, 10]. However, the use of sirolimus alone [15] or in combination with cyclosporine [14] has been associated with an increased risk of developing TMA. When drug-induced TMA is treated with discontinuation of the CNI or PSI alone, the graft loss rate can be 60–100% [6]. It has been reported that belatacept was used successfully as an immunosuppressive drug in transplant patients with *de novo* TMA [8] and this guided our choice of the agent. Belatacept is a primary maintenance immunosuppressive drug that blocks the CD28 costimulation pathway, preventing the activation of T lymphocytes. It is used in combination with other agents to prevent graft rejection in *de novo* renal transplant patients [11, 16]. We used a low-intensity regimen, administered as described above. Also, we used belatacept as if administered *de novo* to achieve an effective immunosuppression regimen with three agents, as is always performed at our center. The use of belatacept does have two main shortcomings that should be considered: its cost and its administration by intravenous infusion. As an adjunct therapy to belatacept introduction and offending drug withdrawal, we used plasmapheresis in one of our patients, following recommendations found in the literature [8].

## 4. Conclusions

To our knowledge, this is the largest series of renal transplant patients with *de novo* drug-induced TMA managed with belatacept as an alternative immunosuppressive drug. The cause of TMA resolution could not be identified because of the multiple and simultaneous factors involved in each case. We acknowledge the limitation of the very short-duration followup in our cases. Although we cannot generalize our results, they support the promising outcomes of previous case reports that belatacept was an effective and safe alternative immunosuppressive agent for the management of renal transplant patients with *de novo* drug-induced TMA.

## Abbreviations

AMR: Antibody mediated rejection
CNI: Calcineurin inhibitors
DSA: Donor-specific antibodies
HUS: Hemolytic uremic syndrome
IVIg: Intravenous immunoglobulin
MPA: mycophenolic acid
POD: Postoperative day
TMA: Thrombotic microangiopathy
PSI: Proliferation signal inhibitors.
